# Improved quality of life after microvascular decompression for hemifacial spasm

**DOI:** 10.1016/j.bas.2026.105987

**Published:** 2026-02-23

**Authors:** Martin E. Weidemeier, Victoria Bogaczyk, Henry W.S. Schroeder

**Affiliations:** Department of Neurosurgery, University Medicine Greifswald, Greifswald, Germany

**Keywords:** Hemifacial spasm, Microvascular decompression, Quality of life, SF-36, Treatment outcomes

## Abstract

**Introduction:**

This study aimed to evaluate the effectiveness of microvascular decompression (MVD) in improving the quality of life (QoL) of patients with hemifacial spasm (HFS), focusing on both short- and long-term outcomes.

**Research question:**

When will patients with HFS benefit from MVD regarding QoL?

**Material and methods:**

A longitudinal, prospective cohort study was conducted, involving 135 patients who underwent MVD at a tertiary referral center between January 2019 and March 2023. Health-related quality of life (HR-QoL) was assessed using the SF-36 questionnaire administered at three intervals: before surgery, three months after surgery, and twelve months after surgery.

**Results:**

Most patients reported complete resolution (63.4%) or at least 90% reduction (13.9%) of spasms after twelve months. Significant improvements were observed in the SF-36 scores, particularly in the domains of Mental Health and Social Functioning, from baseline to twelve months after surgery. Additionally, improvements in Mental Component Summary scores were statistically significant, suggesting substantial importance of patient-reported mental and emotional well-being. Permanent postoperative complications (hearing reduction/loss, mild hoarseness) were seen in 2.9%.

**Discussion and conclusion:**

The findings confirm that MVD provides significant and sustained improvements in HR-QoL in patients with HFS. The surgery not only alleviates physical symptoms but also contributes to substantial psychosocial recovery. These outcomes support MVD as a preferred treatment for HFS when a neurovascular conflict is suspected, advocating for its broader application in clinical practice. Continued follow-up and research are recommended to further document the procedure's long-term effectiveness and safety.

## Highlights

• 63.4% complete spasm resolution; 13.9% achieved ≥90% reduction at 12 months.
• Significant SF-36 improvements in Mental Health and Social Functioning domains.
• Statistically significant Mental Component Summary gains indicate psychosocial recovery.
• Only 2.9% permanent complications (hearing loss/reduction, mild hoarseness).
• MVD demonstrates sustained effectiveness as preferred treatment for hemifacial spasm.

## 1 Introduction

Hemifacial spasm (HFS) is a neurological condition characterized by involuntary, intermittent contractions of the facial muscles on one side of the face. The mean prevalence of the disorder is approximately 10 per 100,000 (Nurminen et al., 2023; Auger and Whisnant, 1990; Nilsen et al., 2004), with specific rates of 11 per 100,000 in females and 7.9 per 100,000 in males (Nurminen et al., 2023). It predominantly affects adults, with a higher incidence among women and individuals over 40 years of age (Nurminen et al., 2023). The progressive nature of HFS can interfere with daily activities such as driving and often leads to social withdrawal due to the stigma associated with visible symptoms and a consecutively reduced health-related quality of life (HR-QoL) (Heuser et al., 2007; Ray et al., 2010; Tan et al., 2005; Lawrence et al., 2018; Rosenstengel et al., 2012).

Microvascular decompression (MVD) offers a definitive treatment by surgically resolving a vascular compression of the facial nerve (Jho and Jannetta, 1987), potentially providing a long-term solution. Despite the risks associated with neurosurgical procedures, there is clinical evidence suggesting that patients generally report substantial improvements in HR-QoL and significant reductions or often even complete resolution of spasms after MVD (Lawrence et al., 2018). This study aimed to prospectively assess the HR-QoL of patients before and after MVD, evaluating its postoperative course over two time points, and analyzing its complications.

## 2 Methods

Patients undergoing MVD for HFS at our department were invited to participate in this prospective study. Participants completed the Short Form-36 (SF-36) questionnaire to assess quality of life at three predefined time points: preoperatively (t1), three months postoperatively (t2), and twelve months postoperatively (t3). Physical and Mental Component Summary scores were calculated using the German population norm from 1994. Scores range from 0 to 100, with higher values indicating better health status.

In addition, participants self-assessed their spasm activity, defining preoperative spasm activity as a baseline value of 100%. Postoperative residual spasm activity was evaluated using a continuous scale ranging from 0% to 100%. For outcome analysis, residual spasm activity was categorized into four outcome groups: 0% residual activity was defined as an excellent outcome, ≤10% residual activity as a good outcome, ≤50% residual activity as a fair outcome, and >50% residual activity as a poor outcome.

Data on demographics, preoperative symptoms, disease duration, MRI findings, and surgical outcomes were collected prospectively. Clinical information came from routine inpatient care. Written informed consent for both surgery and quality-of-life assessment was obtained before intervention. The study received approval from the local institutional ethics committee and was conducted in line with the Declaration of Helsinki (1964). Statistical analyses were carried out with Prism 10.1.1 for macOS (GraphPad Software LLC, San Diego, CA, USA). Continuous variables were checked for normality with the D'Agostino–Pearson omnibus K^2^ test. Depending on distribution, repeated-measures comparisons were done using either parametric two-way ANOVA with Tukey's post hoc tests or the nonparametric Friedman test with Dunn's post hoc tests. Correlations were assessed via Spearman's rank correlation. Categorical data were analyzed with Fisher's exact test. Descriptive results are shown as mean ± standard deviation, along with p-values and 95% confidence intervals. For comparisons, differences, adjusted p-values, and 95% CIs are provided. A p-value of ≤0.05 was deemed statistically significant, corresponding to a 95% confidence level.

## 3 Results

### 3.1 Study cohort and demographics

From January 2019 to March 2023, 135 patients undergoing MVD for HFS were included in the study. Of these, 101 patients provided complete datasets at three evaluation points - before surgery, three months after surgery, and twelve months after surgery - and were included in the analysis. 34 patients were excluded due to incomplete data of SF-36 assessment during follow-up (see Supplementary Table). The cohort predominantly consisted of female patients (67 females vs. 34 males) and showed a higher incidence of HFS on the left side (62 left vs. 39 right). The mean age was 55.5 ± 12.2 years, with an average symptom duration of 7.8 ± 6 years before surgery (see Table 1).

**Table 1:** Summary of Patient Characteristics and Clinical Outcomes 12 months after surgery.

Characteristic	Excellent (100% reduction)	Good (90 – 99% reduction)	Fair (50 – 89% reduction)	Poor (0 – 49% reduction)	Total
n (%)	64 (63.4%)	14 (13.9%)	9 (8.9%)	14 (13.9%)	101 (100%)
Age	55.3 ± 12.1 y	54.1 ± 12.1 y	57.4 ± 10 y	64.8 ± 9.9 y	56.6 ± 12 y
Duration of Symptoms	7.6 ± 6.2 y	8.1 ± 7.9 y	8.7 ± 6.2 y	8.5 ± 5.9 y	7.9 ± 6.3 y
Sex					
Female	54.7%	78.6%	77.8%	100%	66.3%
Male	45.3%	21.4%	22.2%	0%	33.7%
Side					
Left	64.1%	64.3%	44.4%	57.1%	61.4%
Right	35.9%	35.7%	55.6%	42.9%	38.6%
Neurovascular conflict					
AICA	45.3%	28.6%	44.4%	57.1%	44.6%
PICA	65.6%	71.4%	44.4%	35.7%	60.4%
VA	31.3%	14.3%	33.3%	14.3%	26.7%
Grooving of facial nerve	32.8%	57.1%	22.2%	35.7%	35.6%
Postoperative deficits					
immediately	21.9%	7.1%	33.3%	7.1%	18.8%
after 12 months	4.7%	0%	0%	0%	2.9%

HFS outcome refers to the patients' self-evaluated impression of symptom relief 12 months after surgery.

Age and symptom duration are presented as mean ± SD.

Neurovascular conflict refers to the participation of named vessels, and a combination of two or more vessels may have been present.

AICA, anterior inferior cerebellary artery.

PICA, posterior inferior cerebellary artery.

VA, vertebral artery.

### 3.2 Postoperative HFS activity (Fig. 1)

Three months after surgery, patients showed an average residual spasm activity of 20.3% ± 31.4%. The 95% confidence interval was 14.1% to 26.5%. By twelve months, this had decreased to 15.6% ± 30.7% (95% CI: 9.5%–21.7%). Compared with the preoperative baseline, these reductions were statistically significant at both postoperative timepoints (t1 vs. t2, p < 0.0001; t1 vs. t3, p < 0.0001). Still, the difference between the three- and twelve-month points was not significant (p > 0.9999). At one year, 64 patients (63.3%) had complete resolution of spasms. An additional 14 patients (13.9%) showed good improvement, defined as more than a 90% drop in spasm activity. Conversely, 9 patients (8.9%) reported only fair relief, while 14 patients (13.9%) experienced poor relief.

During extended follow-up, 69 patients (68.3%) reported excellent outcomes, 20 (19.8%) good, 6 (5.9%) fair, and 6 (5.9%) poor. The most recent follow-up time was 28.7 ± 16.2 months (range 12–63 months).

### 3.3 SF-36 questionnaire (HR-QoL assessment; Fig. 2)

Physical Functioning: There were no statistically significant changes in mean scores from preoperative baseline score (85.8 ± 21; 95% CI 81.6 – 89.9), at three months (86.4 ± 20.3; 95% CI 82.4 – 90.4), and at twelve months (86.5 ± 22.5; 95% CI 82.1 – 90.9) postoperatively.

Physical Role Functioning: Mean scores significantly improved from preoperative baseline score (63.1 ± 39.2; 95% CI 55.4 – 70.9) to three months (75.3 ± 38.3; 95% CI 67.7 – 82.8) and twelve months (77.7 ± 38.4; 95% CI 70.1 – 85.3) postoperatively (t1 vs. t2 p = 0.0456; t1 vs. t3 p = 0.0131).

Physical Pain: Mean scores remained similar from preoperative baseline score (78.5 ± 27.6; 95% CI 73.1 – 84) to three months (80.5 ± 24.6; 95% CI 75.6 – 85.3) and twelve months (81.7 ± 26.1; 95% CI 76.5 – 86.8) postoperatively, with no statistically significant differences.

General Health: Significant improvements were observed from preoperative baseline score (60.2 ± 21.6; 95% CI 55.9 – 64.5) to three months (66.1 ± 20.2; 95% CI 62.1 – 70.1) and twelve months (68.1 ± 22.2; 95% CI 63.7 – 72.5) postoperatively (t1 vs. t2 p = 0.0118; t1 vs. t3 p = 0.0005).

Vitality: Improved significantly from preoperative baseline score (51.3 ± 19.6; 95% CI 47.4 – 55.1) to three months (62.3 ± 17.3; 95% CI 58.9 – 65.7) and twelve months (63.4 ± 19.5; 95% CI 59.5 – 67.2) postoperatively (t1 vs. t2 p < 0.0001; t1 vs. t3 p < 0.0001).

Social Functionality: Showed significant improvements from preoperative baseline score (63.7 ± 27.9; 95% CI 58.2 – 69.3) to three months (82.8 ± 23.9; 95% CI 78.1 – 87.5) and twelve months (82.8 ± 24.5; 95% CI 78 – 87.6) postoperatively (t1 vs. t2 p < 0.0001; t1 vs. t3 p < 0.0001).

Emotional Role Functioning: No statistically significant changes were observed from preoperative baseline score (68 ± 40.8; 95% CI 59.9 – 76) to three months (84.2 ± 35.5; 95% CI 77.7 – 90.6) and twelve months (83.5 ± 33.5; 95% CI 76.9 – 90.1) postperatively.

Mental Health: Significantly improved from preoperative baseline score (61.8 ± 20.5; 95% CI 57.7 – 65.8) to three months (74.1 ± 16; 95% CI 71 – 77.3) and twelve months (74.6 ± 17.4; 95% CI 71.1 - 78) postoperatively (t1 vs. t2 p < 0.0001; t1 vs. t3 p < 0.0001).

Physical Component Summary (PCS): No significant changes were observed from preoperative baseline score (50.9 ± 9.2; 95% CI 49.1 – 52.7) to three months (49.8 ± 9.5; 95% CI 47.9 – 51.7) and twelve months (50.5 ± 10; 95% CI 48.5 – 52.5) postoperatively.

Mental Component Summary (MCS): Mean scores significantly improved from preoperative baseline score (37.8 ± 14.7; 95% CI 34.9 – 40.7) to three months (48.2 ± 11.5; 95% CI 45.9 – 50.5) and twelve months (48.2 ± 11.9; 95% CI 45.9 – 50.6) postoperatively (t1 vs. t2 p < 0.0001; t1 vs. t3 p < 0.0001).

### 3.4 Comparisons and correlations (Fig. 3)

Gender analysis (Fig. 3C) shows a consistent pattern: across the study, men (n = 34) tended to have higher MCS scores than women (n = 67). At baseline, the difference was statistically meaningful (mean difference 9.1, p = 0.0005, 95% CI 4.1 to 14.2). Three months after surgery, the gap remained (mean difference 7.1, p = 0.0062, 95% CI 2 to 12.2), and at twelve months the difference was still notable (mean difference 7.9, p = 0.0025, 95% CI 2.8 to 12.9). In contrast, PCS scores did not show a significant difference.

**Fig. 1:**
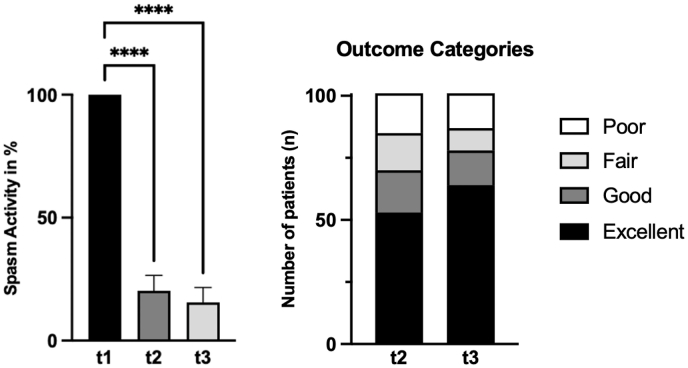
This figure presents the outcomes of 101 patients treated with microvascular decompression (MVD) for hemifacial spasm (HFS) at three time points: preoperatively (t1), three months postoperatively (t2), and twelve months postoperatively (t3). On the left, the graph shows the mean self-assessed HFS activity and standard deviation, with the preoperative baseline set to 100%. There is a significant reduction in HFS activity at t2, with further improvement at t3. On the right, the graph categorizes postoperative outcomes at t2 and t3 into four groups: excellent (0% residual spasm activity), good (10% residual spasm activity or less), fair (50% residual spasm activity or less), and poor (51% residual spasm activity or more). The distribution of patients across these categories demonstrates the increasing effectiveness of MVD over time, with more patients reporting excellent and good outcomes at t3.

**Fig. 2:**
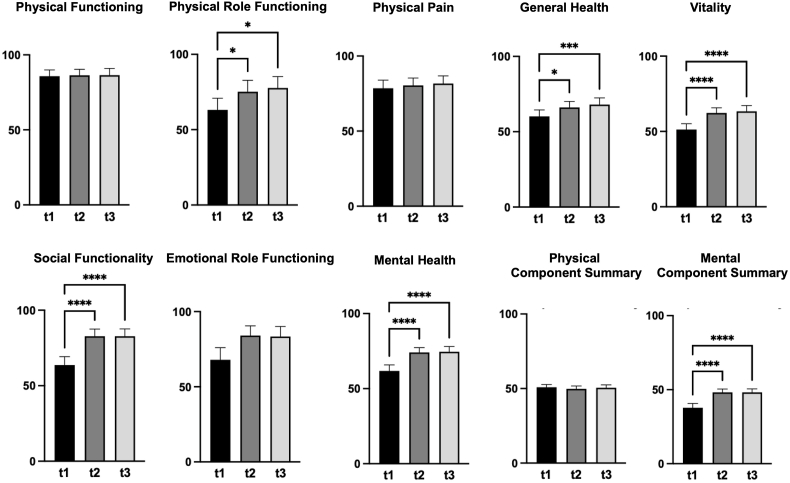
This figure presents the outcomes of 101 patients with microvascular decompression (MVD) for hemifacial spasm (HFS), self-assessing their quality of life via Short-Form 36 (SF-36) at three time points: preoperatively (t1), three months postoperatively (t2), and twelve months postoperatively (t3). Each graph depicts individual domains of the SF-36, which measures various aspects of health-related quality of life. The graphs illustrate the changes in patients' self-reported SF-36 scores across the three time points, showing improvements in various health domains following the surgical intervention. Significant improvements were observed in Physical Role Functioning, General Health, Vitality, Social Functionality, Mental Health and Mental Component Summary. The mean scores and standard deviations for each SF-36 item are displayed.

**Fig. 3:**
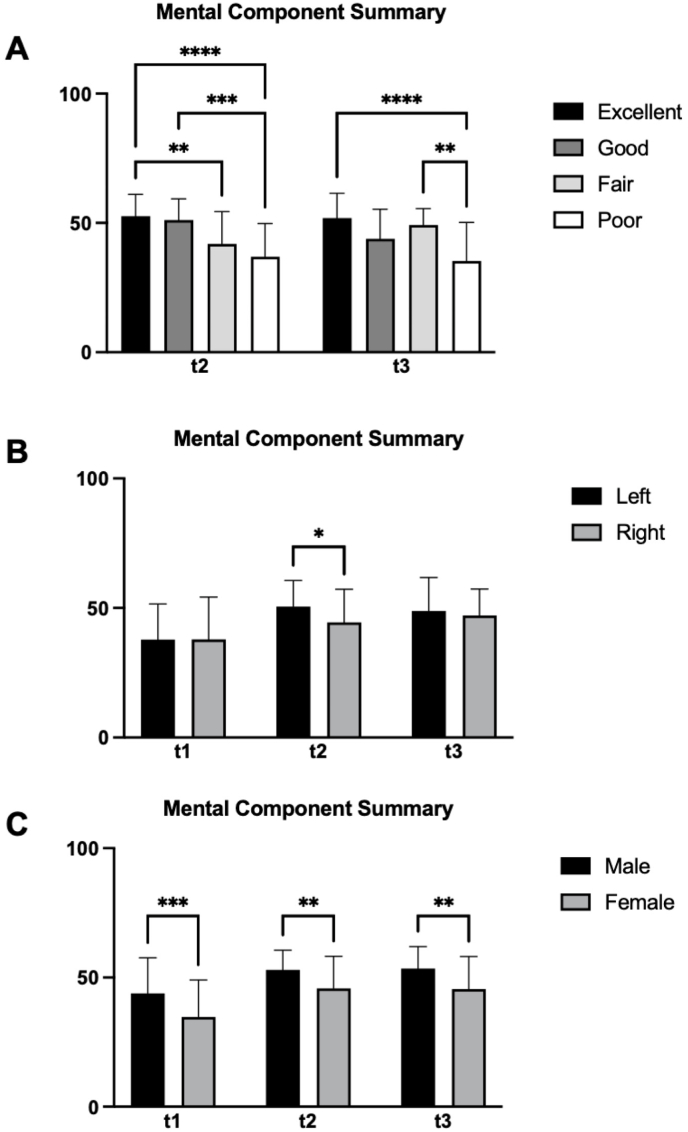
This figure presents a subgroup analysis of the Mental Component Summary (MCS) scores from the Short-Form 36 (SF-36) questionnaire of 101 patients undergoing microvascular decompression (MVD) for hemifacial spasm (HFS) at three time points: preoperatively (t1), three months postoperatively (t2), and twelve months postoperatively (t3). A illustrates the differences in MCS scores between outcome groups (excellent, good, fair, and poor). Patients in the excellent and good outcome groups show significant improvements in their MCS scores at t2 and t3 compared to t1, with the excellent group (complete resolution of spasms) achieving the highest scores. The fair and poor outcome groups also show improvements, however differences are less. B compares MCS scores between patients with left-sided and right-sided HFS. Both groups show similar trends of improvement postoperatively, with mean MCS scores increasing at t2 and t3 while only having a significant difference at t2. C presents the MCS scores for male and female patients. Both genders exhibit significant improvements in MCS scores postoperatively, with mean scores increasing at t2 and t3. Female patients tend to show slightly higher MCS scores compared to male patients at all three time points, though the overall trend of improvement is similar for both groups. Categories: excellent (0% residual spasm activity), good (10% residual spasm activity or less), fair (50% residual spasm activity or less), and poor (51% residual spasm activity or more).

Side of HFS (Fig. 3B): When comparing MCS scores for left-sided (n = 62) versus right-sided (n = 39) HFS, a significant difference emerged at three months postoperatively (mean difference 6.1, p = 0.02, 95% CI 1 to 11.2), with higher MCS among left-sided cases. PCS scores, however, did not differ significantly.

Outcome Categories (Fig. 3A): At three months postoperatively, notable differences in MCS scores were evident among the various outcome categories. Patients with an excellent outcome showed significantly higher scores than those with fair or poor outcomes (excellent vs. fair: mean difference 10.8, p = 0.0092, 95% CI 1.6 – 19.9; excellent vs. poor: mean difference 15.8, p < 0.0001, 95% CI 6.8 – 24.7). A similar pattern was observed when comparing good to poor outcomes (good vs. poor: mean difference 14.3, p = 0.0027, 95% CI 3.2 – 25.3). These differences persisted at twelve months, notably between excellent and poor outcomes (excellent vs. poor: mean difference 16.6, p < 0.0001, 95% CI 7.4 – 25.9) and between fair and poor outcomes (fair vs. poor: mean difference 14, p = 0.032, 95% CI 0.7 – 27.4). No statistically significant difference was observed in comparison of PCS scores.

Duration of Symptoms and Grooving: Patients with a symptom duration of five years or less did not score differently on the MCS compared to those with a longer symptom duration of six or more years, at either three months postoperatively (mean difference 1.2, p = 0.8552, 95% CI -4.1 to 6.4) or twelve months postoperatively (mean difference 4.8, p = 0.0804, 95% CI -0.5 to 10). When examining HFS activity after twelve months, the duration of symptoms of six years or more prior to surgery did not significantly increase the risk of an insufficient outcome (i.e., fair or poor results; Odds Ratio [OR] 1.69, p = 0.3439, 95% CI 0.67 to 4.48). Similarly, a longer symptom duration was not associated with an increased likelihood of finding a groove on the facial nerve intraoperatively (OR 0.85, p = 0.8347, 95% CI 0.38 to 1.87). Additionally, the intraoperative finding of a groove did not significantly predict symptom relief at twelve months postoperatively. Comparing patients with excellent and good symptom relief to those with insufficient relief, the presence of a groove was not a significant factor (OR 0.74, p = 0.6268, 95% CI 0.29 to 1.93).

Correlations: A strong inverse correlation was identified between the MCS and HFS activity at twelve months postoperatively (Spearman r = −0.46, p < 0.0001, 95% CI -0.61 to −0.29), suggesting a significant association between reduced spasm activity and improved MCS. Additionally, a moderate inverse correlation was noted between the MCS and patient age (Spearman r = −0.31, p = 0.0018, 95% CI -0.49 to −0.11). No significant correlations were detected regarding the duration of symptoms before surgery, gender, or affected side with MCS or HFS activity twelve months after surgery.

### 3.5 Complications

No life-threatening complications occurred. However, predominantly transient focal postoperative complications were observed in 19 patients (18.8%) immediately after the procedure. These complications included unilateral deficits involving the cochlear nerve (12 cases), vagal nerve (2 cases), facial nerve (2 cases), vestibular nerve (2 cases), and combined vestibulocochlear nerve involvement (1 case). In 16 of these patients, the complications resolved completely within 12 months, resulting in a long-term postoperative deficit rate of 2.9%. The remaining three patients developed permanent complications: one patient experienced complete unilateral hearing loss, one patient developed partial hearing loss, and one patient had persistent mild hoarseness.

## 4 Discussion

HFS significantly decrease the quality of life of affected individuals. In our patients, MVD improved the quality of life.

### 4.1 Postoperative HFS activity

Our study demonstrates significant relief from HFS in patients undergoing MVD, with the vast majority experiencing sufficient symptom relief. Although most improvements were observed at three months after surgery, 27 patients continued to experience reductions in their spasm activity until twelve months. Delayed cure is a known phenomenon in HFS post-MVD (Nunta-Aree et al., 2024), with up to 12% of patients experiencing relief beyond the one-year mark and occasionally even after three and a half years (Sindou, 2005). Regarding the 14 patients who did not achieve clinically relevant reductions in their spasm activity after 12 months, further improvement during subsequent follow-up was possible. However, even if these patients continue to experience unsatisfactory results, this outcome aligns with other series, where 13-14% of patients did not achieve satisfactory results (Nunta-Aree et al., 2024; Sindou, 2005). Further assessments of our series will be interesting to determine if delayed complete remission in these patients might develop at a later stage.

### 4.2 Quality of life after MVD

The study's results highlight the positive effect of MVD on HR-QoL. While the physical impact of HFS is not the main complaint for most patients, there were clear gains in general health, vitality, social functioning, and mental well-being. The MCS scores rose significantly after MVD, with social functioning showing the largest improvement. This fits with many patients' histories of withdrawing socially due to stigma from uncontrolled facial movements, which are particularly noticeable during speech and emotional expression. As spasms decrease or disappear, patients reported re-engaging in social events again without worrying about embarrassment.

Other studies have also found QoL improvements after MVD in HFS patients, though they used different tools to measure HR-QoL. Our study used the SF-36, an established generic self-report instrument that investigates multiple HR-QoL domains. There are newer, more HFS-specific measures as well, such as HFS-7 (Tan et al., 2005), HFS-8 (Heuser et al., 2007), modified HFS-8 (Lawrence et al., 2018)^,^ (Safonova et al., 2020), or extended HFS-30 (Tan et al., 2004)^,^ (Cheng et al., 2017). However, they lack clinical assessement, which is why some authors have proposed the HFS-Score (Wabbels and Yaqubi, 2021) to bridge this gap by blending objective evaluations with patient-reported severity.

Moreover, our data show a strong inverse relationship between MCS scores and residual spasm activity: fewer spasms align with higher MCS scores. When outcomes were categorized as excellent, good, fair, or poor – following Sindou's personal classification (Sindou and Mercier, 2018) – the best results were achieved in patients with meaningful symptom relief, reflected in significant improvements in MCS. This underscores the profound impact of the disease on daily life and points to MVD as a potentially lasting therapy for spasm-related challenges.

### 4.3 Risk factors

While botulinum toxin injections has been shown to be an effective treatment option for symptom relief (Jitpimolmard et al., 1998), it does not address the mechanical conflict between the vessel and the facial nerve. This ongoing neurovascular conflict leads to permanent irritation of the nerve, which may potentially worsen structural damage over time. Potential signs of the latter may be found in grooving of the facial nerve, which one might expect to be seen predominantly in prolonged disease. However, our analysis found no evidence to support this in our cohort. Thus, a longer duration of spasm activity exceeding five years does not appear to be a risk factor for poorer outcomes one year after MVD. Furthermore, the presence of grooving on the nerve had no prognostic value, which is in contrast to our previous evaluation (Al Menabbawy et al., 2022).

### 4.4 Complications

In our cohort, we observed direct postoperative complications in 18.8% of patients, of which 2.9% persisted twelve months after surgery. The three affected patients with permanent deficits - complete hearing loss, partial hearing loss, and mild hoarseness - expressed in personal conversations that these deficits were less limiting than the initial HFS. They stated that given the complete resolution of their spasms, they would not trade their excellent outcomes for the restoration of these deficits. The complication rate in our cohort aligns with other studies of MVD for HFS (Lawrence et al., 2018; Cheng et al., 2017).

### 4.5 Limitations

The study's findings may be influenced by selection bias since only patients undergoing MVD at our institution were included. As a result, the HR-QoL outcomes might not fully reflect the broader HFS population. The study's design compounds this issue: it took place at a single center, performed by one surgeon, within a specialized tertiary referral center. This setting may decrease generalizability of the results to other institutions.

Another consideration is that the inclusion criteria could have favored patients with more severe symptoms, which might tilt HR-QoL assessments toward those most likely to gain from surgery. While the SF-36 is a widely used tool for general HR-QoL, it may miss some HFS-specific challenges. Implementation of HFS-tailored scoring systems could provide more precise insights into how treatments affect this particular group.

## 5 Conclusions

Our study demonstrated significant improvements in HR-QoL after MVD for HFS, with the benefits of largely satisfactory spasm remission outweighing the low incidence of permanent postoperative focal deficits. Given the psychosocial impacts of HFS, it is critical to maintain a patient-centric approach in treatment decision-making.

## 6 Previous presentations

Parts of the data were presented as part of a talk at the annual German Congress of Neurosurgical Society (Deutsche Gesellschaft für Neurochirurgie, DGNC) on June 26th^,^ 2023 in Stuttgart, Germany as well as part of a oral e-Poster at the annual Congress of the European Association of Neurosurgical Society (EANS) on September 27th^,^ 2023 in Barcelona, Spain.

## Authors contributions:

HWSS and MEW conceived and initiated the study, developing the research goals and the initial project plan. MEW and HWSS collectively designed the methodology and oversaw its implementation. MEW managed the data-collection process, ensuring accuracy and consistency. MEW and VB performed the initial analyses. MEW also took the lead in drafting the manuscript, preparing the original versions, and developing the visual representations of the data. HWSS and VB provided critical reviews, extensively edited the manuscript, and contributed additional information. HWSS also supervised the entire project, guiding the research trajectory and decision-making process. All authors have discussed the results, contributed to the final manuscript, and approved the submitted version.

## Funding

None.

## Declaration of competing interest

The authors declare that they have no known competing financial interests or personal relationships that could have appeared to influence the work reported in this paper.

## Abbreviations:

HFS, Hemifacial spasm; HR-QoL, health-related quality of life; MCS, Mental Component Summary; MVD, microvascular decompression; SF-36, Short-Form 36; PCS, Physical Component Summary.
