# Supplementary material for: Improved quality of life after microvascular decompression for hemifacial spasm
Source: Brain Spine. 2026 Feb 23;6:105987. doi: 10.1016/j.bas.2026.105987 (PMC12955192; doi:10.1016/j.bas.2026.105987)
Supplement: Multimedia component 1 [file mmc1.docx]

Supplementary Table 1

Summary of excluded Patients (Characteristics and Clinical Outcomes)

|  | Excellent (100% reduction) | Good  (90 – 99% reduction) | Fair  (50 – 89% reduction) | Poor  (0 – 49% reduction) | Total *  (n = 34) | SF-36-cohort  (n = 101) |
| --- | --- | --- | --- | --- | --- | --- |
| Outcome  3 months postoperatively (t2)  n (%) | 9  (26.5%) | 6  (17.6%) | 6  (17.6%) | 1  (2.9%) | 22 **  (64.7%) |  |
| Outcome  12 months postoperatively (t3)  n (%) | 10  (29.4%) | 4  (11.8%) | 0  (0%) | 2  (5.9%) | 16 **  (47.1%) |  |
| Characteristic *** | | | | |  |  |
| Age | 48.7 ± 12.2 y | 45 ± 9.2 y |  | 61 ± 12.7 y | 51.7 ± 12 y | 56.6 ± 12 y |
| Duration of Symptoms | 8.6 ± 4.6 y | 6.8 ± 4.9 y |  | 12 ± 5.7 y | 7.7 ± 4.7 y | 7.9 ± 6.3 y |
| Sex |  |  |  |  |  |  |
| Female | 20% | 50% |  | 100% | 47.1% | 66.3% |
| Male | 80% | 50% |  | 0% | 52.9% | 33.7% |
| Side |  |  |  |  |  |  |
| Left | 90% | 100% |  | 0% | 59.4% | 61.4% |
| Right | 10% | 0% |  | 100% | 40.6% | 38.6% |
| Neurovascular conflict |  |  |  |  |  |  |
| AICA | 40% | 50% |  | 100% | 58.1% | 44.6% |
| PICA | 50% | 25% |  | 0% | 45.2% | 60.4% |
| VA | 10% | 25% |  | 0% | 9.7% | 26.7% |
| Grooving of facial nerve | 30% | 25% |  | 50% | 26.5% | 35.6% |
| Postoperative deficits |  |  |  |  |  |  |
| immediately | 10% | 25% |  | 0% | 8.8% | 18.8% |
| after 12 months | 0% | 0% |  | 0% | 0% | 2.9% |

Patients were excluded due to incomplete SF-36 assessments at either 3 months or 12 months after surgery.
HFS outcome refers to the patients’ self-evaluated impression of symptom relief 3 months and 12 months after surgery.

Presented epidemiological data refers to categorization

* of 5 patients no postoperative information was available due to immediate loss to follow-up. Data in column refers to all excluded patients (n = 34).

** inconsistent number due to incomplete assessments / loss to follow-up

*** Data shown refers to categorization of available data at 12 months after surgery (consistency to Table 1)

Age and symptom duration are presented as mean ± SD.

Neurovascular conflict refers to the participation of named vessels, and a combination of two or more vessels may have been present.

AICA, anterior inferior cerebellary artery

PICA, posterior inferior cerebellary artery

VA, vertebral artery
